# Sympathetic nerve activity in normal and cystic follicles from isolated bovine ovary: local effect of beta-adrenergic stimulation on steroid secretion

**DOI:** 10.1186/1477-7827-9-66

**Published:** 2011-05-16

**Authors:** Alfonso H Paredes, Natalia R Salvetti, Ariel E Diaz, Bibiana E Dallard, Hugo H Ortega, Hernan E Lara

**Affiliations:** 1 Laboratory of Neurobiochemistry, Department of Biochemistry and Molecular Biology, Faculty of Chemistry and Pharmaceutical Sciences, Universidad de Chile, Santiago, Chile; 2 Morphological Sciences Department, Faculty of Veterinary Sciences, Universidad Nacional del Litoral (FCV-UNL), Esperanza, Santa Fe, Argentina & National Council for Science and Technology (CONICET), Argentina

## Abstract

Cystic ovarian disease (COD) is an important cause of abnormal estrous behavior and infertility in dairy cows. COD is mainly observed in high-yielding dairy cows during the first months post-partum, a period of high stress. We have previously reported that, in lower mammals, stress induces a cystic condition similar to the polycystic ovary syndrome in humans and that stress is a definitive component in the human pathology. To know if COD in cows is also associated with high sympathetic activity, we studied isolated small antral (5mm), preovulatory (10mm) and cystic follicles (25mm). Cystic follicles which present an area 600 fold greater compared with preovulatory follicles has only 10 times less concentration of NE as compared with small antral and preovulatory follicles but they had 10 times more NE in follicular fluid, suggesting a high efflux of neurotransmitter from the cyst wall. This suggestion was reinforced by the high basal release of recently taken-up ^3^H-NE found in cystic follicles. While lower levels of beta-adrenergic receptor were found in cystic follicles, there was a heightened response to the beta-adrenergic agonist isoproterenol and to hCG, as measured by testosterone secretion. There was however an unexpected capacity of the ovary in vitro to produce cortisol and to secrete it in response to hCG but not to isoproterenol. These data suggest that, during COD, the bovine ovary is under high sympathetic nerve activity that in addition to an increased response to hCG in cortisol secretion could participate in COD development.

## Background

Cystic ovarian disease (COD) is an important cause of abnormal estrous behavior and infertility in dairy cows. The prevalence of COD in dairy herds has been reported to vary from 5 to 30% [1] and this condition may result in significant economic losses to the dairy industry due to increased calving to conception and inter-calving intervals [2] COD is mainly observed in high-yielding dairy cows during the first month's post-partum, as this is a period of high stress [2]. We have previously reported that in lower mammals both the steroid-induced increase in sympathetic nerve activity and stress are able to induce a cystic condition similar to the polycystic ovary syndrome (PCOS) in humans [3-5]. Many possibilities have been involved in human polycystic ovary syndrome (PCOS) development. It has been demonstrated a correlation between high sympathetic nerve activity and PCOS in human [6]. Both hypothalamic and local intraovarian mechanisms have been suggested [7]. Our group has presented evidences that the cold-stress which principally activates sympathetic nerve without modifying corticoids levels [8], could be other of the mechanism involved in the development of follicular cyst in rats [9]. In addition, it has been demonstrated that the effect of stress in the pathogenesis of polycystic ovary PCO is mediated by sympathetic discharge originating at the paraventricular nucleus [10-12]. Therefore, changes in sympathetic nerve activity are a major factor contributing to the changes in sympathetic tone during stress. Regarding this observation, it has also been proposed that sympathetic activity could be a component in bovine COD because stress induces changes in beta-adrenergic receptors at the level of the hypophysis as well as that of the ovary [13]. To analyze the hypothesis that sympathetic nerves are also a principal component in cystic ovarian disease in cows, and because sympathetic nerves penetrate to the mammalian ovary [14,15] to directly innervate theca cells of follicles and the interstitial gland [16], we used a recently published technique to isolate follicles from the cow ovary [17]. We separated small antral (SA), preovulatory (PO) and cystic follicles to analyze the in vitro NE release capacity of sympathetic nerves arriving at specific follicular compartments in the ovary and the related neurogenic-dependent steroidal secretory response. Our results strongly suggest that sympathetic activity is one of the components in maintaining the increased secretory activity associated with the development of COD in cows.

## Methods

### Collection and preparation of tissues

Ovaries with normal morphology (n = 16) and with spontaneous cystic follicles (n = 16) were collected at a local abattoir, within 20 min of death, from mixed breeds of *Bos taurus *cows that were assessed visually as being non-pregnant and without macroscopic abnormality in the reproductive system, Based on the records contributed by the veterinary inspection, the animals were in the second half of the lactation. The complete ovaries were washed, refrigerated and transported immediately to the laboratory. The surgical procedure used to obtain the SA, PO and a cystic follicle has recently been described [17]. During dissection, the follicular diameter was measured with calipers and follicular fluid from each follicle was aspirated and stored separately at -20°C. Only one follicular structure type was obtained from a single ovary collected per animal and follicular health status was confirmed by measurement of hormone concentrations in follicular fluid and morphological analysis [18,19].

Pieces of ovaries from SA, PO and cystic follicles were fixed in 4% paraformaldehyde, embedded in paraffin and cut into 6 μ m sections, then stained with hematoxylin and eosin. The presence of preantral, small antral (SA), preovulatory (PO) and cystic follicles was analyzed according to [5]. Briefly, antral follicles were those in which the nucleus of the oocyte could be visualized. Preovulatory follicles have an average diameter of > 10 mm [1]. Ovarian cysts are defined as follicle-like structures of diameter greater than 25 mm in the absence of a corpus luteum [20]. Our previous observations in rats suggest the existence of a transitional stage between healthy preovulatory follicles and the cystic follicles described previously [5].

### Measurement of NE content

We homogenized 50 mg pieces of follicular wall (SA, PO and cystic) from the ovaries of each animal in 250 μ l DPBS. The homogenized tissue was precipitated with 4 volumes of 0.25 N perchloric acid and centrifuged (15,000 × g, 15 min). We resuspended 50 μ l of follicular fluid in 150 μ L of DPBS, and 100 μ L from this suspension was precipitated with 4 volumes of 0.25 N perchloric acid and centrifuged (15,000xg, 15 min). NE was measured in the acid supernatant by HPLC coupled with electrochemical detection as previously described in [21]. Briefly, 20 μ l of the resulting supernatant (follicular wall or follicular fluid) were injected into a Waters HPLC system equipped with a C18 reverse phase column (Lichrosphere, 60 RP-Select B, Merck, Darmstadt, FR Germany) and an electrochemical detector (Waters 464). The mobile phase contained 0.1 M NaH2PO4, 0.42 mM octyl-sulphate, 0.02% EDTA and 1.5% acetonitrile (pH 2.5) with a 0.9 ml/min flow rate. The potential of the amperometric detector was set to 0.7 V. Under these experimental conditions, the retention time was 4 min for NE and 10 min for DHBA.

### Uptake and release of NE

The procedure used, with some modifications, has previously been described [22,23]. The SA, PO or cystic follicles wall pieces (about 50 mg) were rapidly removed from the ovary and dissected as previously described [17]. Tissues were preincubated for 20 min in Krebs ringer bicarbonate buffer (KRB), pH 7.4, and gassed with 95% O_2 _- 5% CO_2_. Samples were then incubated for 30 min at 37°C with 2 μ Ci ^3^HNE (New England Nuclear Life Science Products, Boston, MA). After incubation, tissues were washed six times, 10 min each, to eliminate radioactivity that was not incorporated into the tissue. After washing the tissue to remove non-incorporated radioactivity, the tissues were placed in a multiwell plate with 24 flat-bottom wells containing 2 ml of buffer per well. Tissues were incubated for 2 min in each well. After 3 passages, depolarization was effected by removal of the tissue to another well with 80 mM K^**+ **^KRB. After stimulation, tissues were washed 3 times (2 min each). At the end of the experiment, the follicular walls were homogenized in 0.4 N perchloric acid, and the level of [^3^H]catecholamines remaining in the tissue were determined by scintillation counting (Tri-Carb Liquid Scintillation Analyzer 1600TR Packard Instruments, Meriden, CT); we obtained 72.5% efficiency for ^3^H in calculating the radioactivity remaining in the tissue after the experiment. The radioactivity incorporated by the tissue and the radioactivity released during stimulation were then calculated. The latter, which represents [^3^H]NE overflow from tissue, was expressed as fractional release, i.e., as a percentage of the total radioactivity present in the tissue [23,24]. The total amount of NE released under stimulation (net release) was calculated as the area under the stimulation minus the spontaneous release [23,25].

### β-adrenergic receptor binding

Membranes were prepared from ovarian follicular wall (pieces of about 50 mg each) by differential centrifugation [3] with minor modifications. In brief, tissues were homogenized in 0.02 M Tris/HCI and 0.25 M sucrose (pH 7.4), and the homogenates were centrifuged at 30000 xg for 20 min. The resulting pellets were suspended in the same buffer and centrifuged as above. This procedure was repeated again, and the pellets were suspended in 0.02 M Tris/HC1 and 10 mM MgCl_2 _(pH 7.4, assay buffer) and then used in the radioreceptor assay. The assay contained a 20 nM saturating concentration of ^3^H-dihydroalprenolol (92.0 Ci/mmol, Dupont/NEN) with membranes (20 μ g of protein) in a total volume of 0.2 ml. Non-specific binding was assessed in tubes containing 10^-4 ^M DL-propranolol. Results are expressed as femtomoles (fmol) of dihydroalprenolol bound/milligram of protein per 30 min at 37ºC. Binding was terminated by the addition of 10 volumes of assay buffer and vacuum filtration through Whatman (Clifton, NJ) GF/C fiberglass as described (Barria et al., 1993). Radioactivity retained on the filters was determined by scintillation counting (Tri-Carb Liquid Scintillation Analyzer 1600TR Packard Instruments, Meriden, CT) with a 72.5% efficiency for ^3^H.

### "In vitro" testosterone and cortisol release

Steroid response to adrenergic and/or gonadotropin stimulation was done by incubating tissue in 2 ml Krebs-Ringer bicarbonate buffer, pH 7.4, for 3 h at 37ºC [26,27], in the presence of D,L-isoproterenol-HCl (10^-5 ^M; Sigma Chem Co., St. Louis, MO), hCG (2.5 IU; Sigma Chem Co., St Louis, MO), or with no stimulation (basal release). The experimental design was such that all ovarian tissues were simultaneously used; one served as a control, and the other two were subjected to the different stimulatory treatments. Testosterone and cortisol released into the incubation medium were measured by ELISA, as previously described [4].

### Hormone assays

Follicular fluid and culture medium, testosterone and cortisol were measured by ELISA kits (Testosterone EIA, DSL-10-4000; Cortisol EIA, DSL 10-2000; Diagnostic Systems Laboratories, Webster, TX), according to the manufacturer's instructions. Testosterone and cortisol concentrations in small follicles were not assayed due to the insufficient volume of follicular fluid collected from these follicles. The assay sensitivity was 0.04 ng/ml for testosterone and 0.1 μ g/dl for cortisol.

The follicular health status was confirmed by measuring the hormonal levels in the follicular fluid. Oestradiol and progesterone in the follicular fluid were measured using ELISA kits (Estradiol EIA, DSL-10-4300; Progesterone EIA, DSL-10-3900; Diagnostic Systems Laboratories, Webster, Texas, USA), according to the manufacturer's instructions. The assay sensitivity was 7 pg/ml for oestradiol and 0.13 ng/ml for progesterone. All follicles were categorized as oestrogen active without luteinization (Table 1).

**Table 1:** Estradiol and progesterone in the follicular fluid

Steroid (ng/mL)	SA	PO	Cyst
Estradiol	97.31 ± 6.65	282.41 ± 22.1*	313.09 ± 18.62*
Progesterone	62.12 ± 9.01	81.52 ± 9.48	57.28 ± 13.53

Results correspond to Mean ± SEM concentrations of 4 determinations for estradiol and progesterone in the follicular fluid of SA, PO and cystic follicles. * = P < 0.05 vs. SA)

### Statistical analysis

Differences between the different ages groups were analyzed by one-way ANOVA, followed by the Student-Newman-Keuls multiple comparison test for unequal replication. The level of significance was set to P < 0.05.

## Results

### Morphological aspects of small antral (SA), preovulatory (PO) and cystic follicles

**Figure 1:**
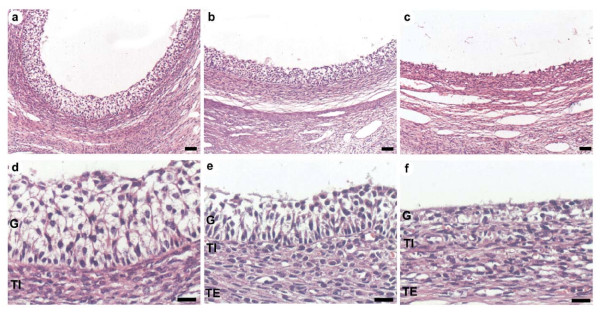
**Microphotography of the follicular wall**. Panel a and d correspond to low and higher magnification of the small antral follicle wall. Panels b and e represent preovulatory follicle wall. Panels c and f are representatives images of the cystic follicle wall. In a, b, and c, bars correspond to 50 μ m, and for d, e and f, bars = 20 μ m.

The morphological characteristics of the ovarian follicles from normal ovulating cows and those with ovarian cystic disease are shown in Figure 1. Small antral (SA) follicles (diameter < 5 mm) and preovulatory (PO) follicles (diameter > 10 mm) have a well-defined granulosa cell layer and internal theca cell layer. Cystic follicles (diameter > 25 mm) obtained from the ovaries of non-ovulatory cows presented a small granulose cell layer, which was basically a monolayer, and a much higher diameter (and thus more volume of follicular fluid) than PO and SA follicles. In a typical experiment, we obtained 30 μ l from each SA, 1 ml from PO follicles and 3 ml from cystic follicles. Due to the substantial difference in the amount of follicular fluid, we decided to analyze all results in terms of concentration and amount separately for each follicular type.

### NE concentration in follicular fluid and in the walls of SA, PO and cystic follicles

**Figure 2:**
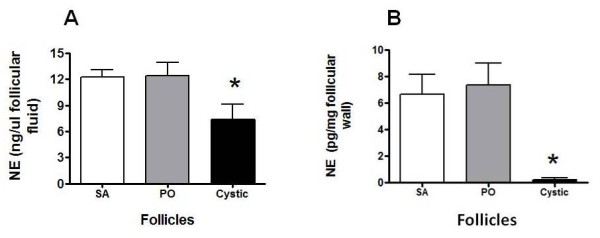
**Concentration of noradrenaline in ovarian follicular fluid and follicular wall**. In A is shown the concentration of the noradrenaline content in follicular fluid and B is shown the concentration in the follicular walls, as well as in the small antral (SA), preovulatory (PO) and cystic follicular wall (cystic). Values represent mean value ± SEM of the number of samples with four individual follicles per experimental group, *, p < 0.05 vs. SA and PO.

We measured NE concentration in each type of follicle (Figure 2B). Although there was no difference between SA and PO follicles, there was a > 90% decrease in NE concentration in the walls of the follicular cysts. To determine whether this difference was due to a change in the compartmentalization of NE, we also measured the NE concentration in the follicular fluid (Figure 2A). There was no difference in NE concentration (expressed as ng NE/μ l of follicular fluid) in follicular liquid obtained from SA, PO and cystic follicles.

### ^3^H-NE uptake and its induced release from SA, PO and cystic follicles

**Figure 3:**
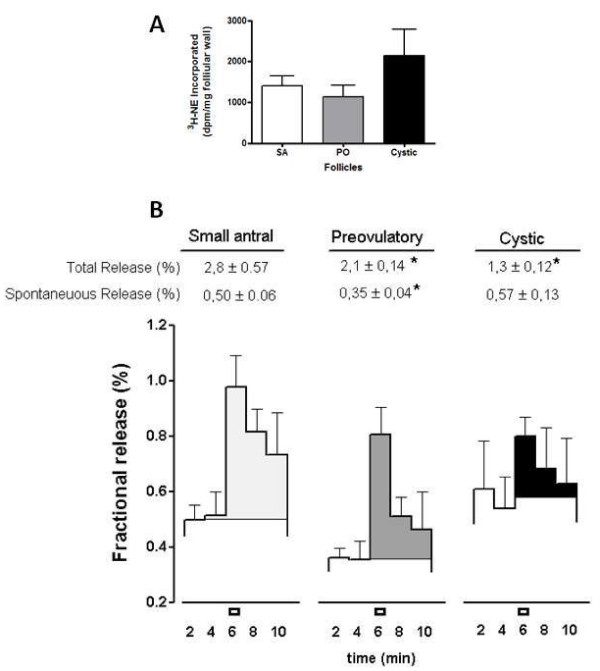
**Incorporation and release of ^3^H-NE from small antral (SA), preovulatory (PO) and cystic follicular wall**. In A is shown the amount of 3H-NE incorporated in follicle wall, In B is shown the release of ^3^H-NE induced by high potassium depolarization (black rectangles). In A results are expressed as dpm per 50 mg tissue and in B the release is expressed as a percentage of ^3^H-NE retained in the tissue at each interval studied. The first row upper numbers represent the total release of ^3^H-NE induced by potassium depolarization and the lower row represents the spontaneous release as percentage of radioactivity released before the depolarization stimulus. *= p < 0.05 vs Small antral (SA) and Preovulatory; & = p < 0.05 vs cystic,

No changes in the amount of ^3^H-NE incorporated and retained by the pieces of follicular wall of the different types of follicles were found (Figure 3A) ). The three types of follicles were able to incorporate NE, and they were also able to release NE when a depolarizing stimulus was applied to the preparation (Figure 3B). There was however a decrease of 54% in the amount of ^3^H-NE released from cystic follicles as compared with SA and PO follicles. It is also interesting to note that basal release, i.e., ^3^H-NE released spontaneously without stimulation, was 37% higher than in PO follicles.

### Changes in β-adrenergic binding sites in small antral, preovulatory and cystic follicles

**Figure 4:**
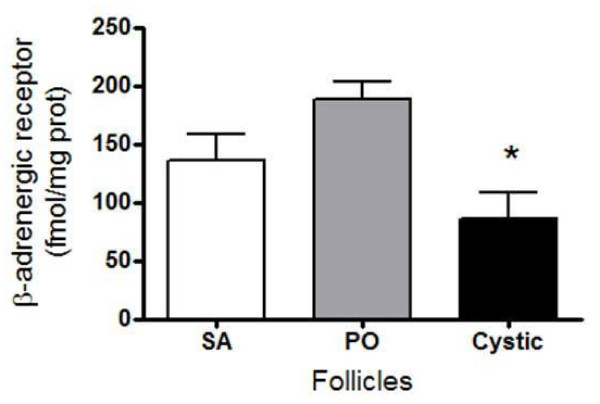
**Concentration of β-adrenergic receptor from small antral (SA), preovulatory (PO) and cystic follicular wall**. The β-receptor concentration is expressed as fmol of dihydroalprenolol bound/mg of membrane protein. Each bar represents the mean ± SEM of 4 experiments, for each group. * = p < 0.01 vs. preovulatory follicular wall.

We determined the total amount of binding sites by incubating tissue with a 20 nM saturating concentration of ^3^H-dihydroalprenolol (Figure 4). The highest number of binding sites (expressed per mg of protein) was found in PO follicles; cystic follicles presented the lowest number.

### Changes in the in vitro release of testosterone and its relation to intrafollicular testosterone concentration

**Figure 5:**
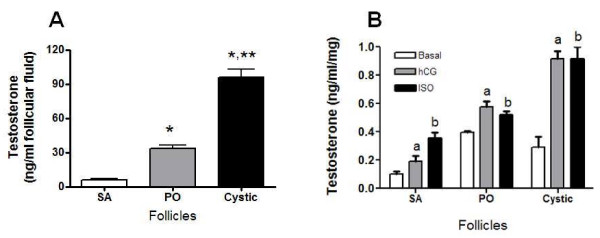
**Testosterone concentration in the ovarian follicular fluid and testosterone secreted *in vitro *in follicular wall**. In A is shown the testosterone concentration in the follicular fluid and in B is shown testosterone secreted following stimulation with a β-receptor agonist (isoproterenol) and with hCG (B). Follicular wall pieces were incubated for 3 h in 2 ml of Krebs-Ringer bicarbonate buffer (basal), 10 μ M isoproterenol (ISO) or 5 IU hCG. Results are expressed as ng testosterone per ml of incubation medium per mg of tissue during 3 h. Each bar represents the mean ± SEM of four independent observations per group. *= p < 0.01 vs Small antral (SA); **= p < 0.01 vs Preoulatory follicles (PO). a, b = p < 0.05 vs. basal (SA, PO and Cystic).

Cysts displayed the highest basal release of testosterone as compared with SA and PO follicles (Figure 5A). Cysts also had the highest responsiveness to both hCG and isoproterenol as measured by testosterone release capacity, in comparison to both SA and PO follicles. To verify whether the increased basal release of testosterone reflects the local synthesis of testosterone, we measured the concentration of testosterone in follicular fluid (Figure 5B). The highest concentration of testosterone was found in follicular cysts; the lowest concentration was observed in SA follicles.

### Changes in the in vitro release of cortisol and its relationship to intrafollicular cortisol concentration

**Figure 6:**
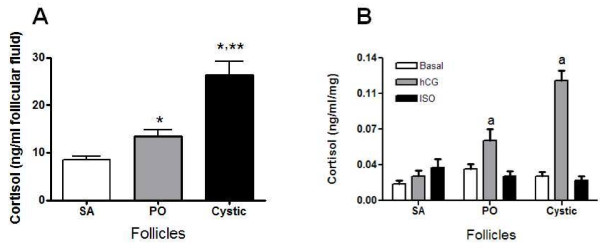
**Cortisol concentration in the ovarian follicular fluid and secreted *in vitro *in follicular wall**. In A is shown the cortisol concentration in follicular fluid and In B is shown of cortisol secreted following stimulation with β-receptor agonist (isoproterenol, 10 uM) and with 5 IU hCG. Follicular wall slides were incubated for 3 h in Krebs-Ringer bicarbonate buffer (basal), 10 uM isoproterenol (ISO) or 5 IU hCG. Results are expressed as ng cortisol per ml of incubation medium per mg of tissue. Each bar represents the mean ± SEM of four independent observations per group. * = p < 0.01 vs Small antral (SA); **= p < 0.01 vs Preoulatory follicles (PO). a= p < 0.05 vs. basal (PO and Cystic).

To verify whether the ovary is strongly influenced by cortisol following stress, we measured the intrinsic capacity of the ovary in vitro to produce cortisol, and we also measured the cortisol concentration in follicular fluid. The highest concentration of cortisol in the follicular fluid was found in cystic follicles; this concentration was twice that found in PO follicles (Figure 6A). There was no change in basal cortisol release among the various follicle types (Figure 6B). PO and especially cystic follicles are highly responsive to hCG stimulation in vitro; however, isoproterenol was not able to elicit any secretory cortisol response from the three types of follicles (Figure 6B).

## Discussion

In this paper, we wanted to analyze the participation of sympathetic nerve activity in the formation and steroid secretory activity of ovarian cysts in dairy cows. As it has described previously in other species, including humans [3,4,6]), we found an increase in the capacity of the cyst to release NE recently taken up and also in the basal NE release suggesting that it could be associated to an increased sympathetic nerve activity and be a component in the COD found in dairy cows. If it is assumed that the changes in the uptake and basal and induced release of NE represent nerve activity, thus the changes in these experimental values could be related to changes sympathetic nerve activity to correlate to ovary function [9]. Many similarities have appeared when comparing rats, human and cows regarding morphometry of ovarian cysts. Morphological aspects of small, preovulatory and cystic bovine follicles are similar to the ones described for others species such as rats and humans [5,7]. In dairy cows, preovulatory follicles have an average diameter of 16-19 mm [1]. Ovarian cysts are defined as follicle-like structures of diameter greater than 25 mm persisting for at least 10 days in the absence of a corpus luteum [20]. A cystic follicle is also characterized by a large accumulation of fluid; the wall of the cyst has a rich vasculature, even more so than that of healthy preovulatory follicles [28]. Recently we used [17] a surgical method to isolate ovarian follicles of different diameters and hence different stages of maturation. Notably, there was a substantial difference in size between the different follicles, especially the cystic follicles. Not only the amount of follicular liquid inside cystic follicles is many times that found in SA follicles, but also the area of follicular wall is highly increased in cystic follicles. Thus, is important to consider these variables to understand the relative contribution of nerve terminals in the piece of follicular wall used in the study. Cyst follicles could be a reservoir of substances secreted by granulose or theca cells, as well as substances permeating from plasma.

The measurement of NE concentration in follicular wall represents the amount of neurotransmitter in close contact to beta-receptors present in both theca and granulosa cells [29,30]. The follicular walls from SA and PO follicles did not differ in NE concentration, but the cyst wall presented a much lower concentration of NE as compared with both SA and PO follicles. As discussed above, the relative decrease in NE concentration could be the result of the big increase in the area of cystic follicles. According to diameter of each type of follicles and because we used follicular pieces of similar size, the relative area used from each follicle is highly variable. (0.25, 25 and 15,625 mm^2 ^from SA, PO and cystic follicles respectively), thus an increase of almost 600 fold between PO and cystic follicles, suggesting not only a higher release activity of the nerves but also an increased efflux of NE from cystic wall to follicular fluid as compared to the efflux existing in SA and PO follicles. In support of this hypothesis, there was a 10-fold increase in the total amount of NE present in follicular fluid obtained from cysts as compared with the follicular fluid derived from PO follicles. Thus it was evident that total NE release from the cystic wall was increased as compared with SA and PO follicles. It was also evident that the cystic wall presented the highest spontaneous release of NE without a change in the capacity to incorporate NE into the tissue. If we consider the increase in spontaneous release together with the induced release, we get a higher release capacity from the cystic follicles as compared to the follicular wall from PO follicles.

Despite of the higher total release of NE (after correction by the increase in the size of cyst), the amount of NE in the cystic follicular fluid seems to exceed the capacity to release NE from the follicular wall of cyst. This contrasts with the polycystic condition induced in rats after estradiol valerate administration in which there is no increase in the size of the ovary and there is a direct correlation between NE amount and release after estradiol treatment [31]. In cows, there is likely an additional factor that it is not present in the estradiol-induced rat model that decreased the stimulus-induced release capacity of the nerve terminals associated with cysts. Stress-induced cortisol release could be one of these factors, as it has been previously described for the rat under a combined cold and restraint stress procedure, in which there is a increase in corticosterone plasma levels [32]. As we discussed below, cystic follicles present an increased capacity to secrete cortisol, either basal or induced by hCG and this hormone could act locally to decrease NE release [32]. Although, the cystic follicles posses few granulosa cells, we have previously reported that granulosa cells have the capacity to incorporate and release NE as a neuron-like cells [33] and thus they could participate as a intrafollicular regulatory compartment for NE. This mechanism required futures studies. Unpublished data give us information that in the rat ovary interstitials cells participate in NE uptake too. The relative contribution of each compartment it is not exactly known but denervation studies [15], strongly suggest that extrinsic innervations (nerve terminals located close to theca cells and in interstitial tissue) correspond at least 80% of total NE. Whatever the underlying mechanism, the continuous non-regulated outflow of NE to the follicular fluid likely induced a decrease in the number of β-adrenergic receptors in the cystic wall as a consequence of a ligand-induced downregulation of the receptor [3,25,32,34]. It has been well documented that β-receptor number in many tissues, including the rat ovary under polycystic conditions [3], is downregulated by increased amounts of NE in the synaptic space, as observed in the follicular cyst. Odore et al., [35] were the first to demonstrate a decrease in β-adrenergic receptor number in follicular cysts from dairy cows. Independently of the decrease in cystic follicle β-receptor number found in this work, we sought to determine whether there is a physiological coupling between β-adrenergic stimulation and steroid secretion from the ovary. The follicular cyst presented a higher capacity to spontaneously release testosterone when incubated in vitro. This characteristic could be in line with an increased sensitivity to NE stimulation derived from hypersensitivity among the low number of β-adrenergic receptors present in the wall of follicular cysts. The continuous stimulation by NE present in the follicular fluid could be responsible for this spontaneous secretion of testosterone. The increased capacity of the piece of follicular cyst to respond to isoproterenol strongly suggests that follicular cysts in dairy cows are highly responsive to β-adrenergic stimulation and induce an elevation in testosterone secretion. We have demonstrated previously that, not only in rats but also in the human ovary [3,9], β-adrenergic stimulation controls androgen secretion and thus participates in the control of steroid secretion. The relationship between increased steroid secretion and stress has been demonstrated in rats as well as in humans [22,36]. In humans it has been demonstrated that women with PCOS present a higher stress level and higher levels of sympathetic nerve activity [6,22]. Anxiety could be also a component of this response to stress [37] and cortisol regulate this response. The higher concentration of cortisol in follicular cysts as compared to SA or PO follicles and the increased response to hCG, introduces the interesting possibility that intrafollicular cortisol could represent an index for the plasma levels of corticoids or even local production by the follicular wall. The in vitro incubation of follicles under stimulation by hCG or isoproterenol clearly demonstrates that hCG but not ß-adrenergic agonist, induced the synthesis of cortisol, leading to the concept that cortisol could be preferentially regulated by a hormonal way (i.e. gonadotropins) and testosterone secretion is regulated by both hormonal and neural stimulation. More studies are needed to characterize the role of cortisol in the context of ovarian function.

Follicular cysts from the ovaries of cows and their pathophysiology are of increasing interest due to the use of β_2_-adrenergic compounds to improve the performance of meat-producing animals [38-40]. β2-adrenergic agonists are powerful compounds that, following long-term administration at high doses, cause a significant repartition of feed energy, thus increasing protein accretion at the muscular level, which results in lipolytic effects. Independent of the possible effects of residues consumed by humans, β-agonists could also be dangerous for the animal's welfare.

## Competing interests

The authors declare that they have no competing interests.

## Authors' contributions

NRS: participated in ovarian morphology and quantification of steroid hormone. AED: chromatographic determination of noradrenaline in ovarian follicular fluid and wall. BED: determination of testosterone concentration and secretion study *in vitro*. HHO: participated in the incorporation and release noradrenaline, secretion study *in vitro*, determination of cortisol an testosterone concentration. HL: discussion and manuscript preparation. AP: participated in the incorporation and release noradrenaline, determination of β-adrenergic receptor in the follicular wall and contributed to the development, design, coordination of the research, manuscript preparation. All authors read and approved the final manuscript.
